# Rapid Development of Autoimmune Hepatitis Secondary to Minocycline

**DOI:** 10.7759/cureus.23038

**Published:** 2022-03-10

**Authors:** Omar Rafa, Eric J Basile, Elisabeth Frankini, Ammar Ahmed

**Affiliations:** 1 Medicine, Touro College of Osteopathic Medicine, New York City, USA; 2 Internal Medicine, Touro College of Osteopathic Medicine, New York City, USA; 3 Medicine, New York Institute of Technology College of Osteopathic Medicine, Old Westbury, USA

**Keywords:** hepatitis workup, steroid therapy, gastroenterology, minocycline, autoimmune hepatitis

## Abstract

Autoimmune hepatitis (AIH) is a condition that affects the liver which, potentially, may render it fibrotic and eventually cirrhotic. This condition has many etiologies ranging from genetic predispositions and immunological defects to medication and environmental side effects. Essentially, we will explore the risks, presentation, diagnosis, and treatment of this condition as it relates to a medication-induced etiology. Here we report a case where a patient developed this condition from taking the antibiotic minocycline. The patient was treated with prednisone therapy and went into complete remission with no reoccurrence of AIH. The purpose of this case report is to highlight the fact that these cases have the potential to occur fairly sooner than expected, in a matter of weeks or months, after the induction of minocycline. Hence, carefully monitoring liver functions more frequently may aide in the prevention of minocycline-induced AIH.

## Introduction

There are two main types of autoimmune hepatitis (AIH). Type 1 accounts for the majority of cases, representing 96% of the total AIH cases in North America [1]. Antinuclear antibodies (ANAs) and anti-smooth muscle antibodies (ASMAs) are the markers for this type. The latter is the more specific marker as the former occurs in many other conditions. Type 2 AIH is more severe and less common accounting for 4% cases in North America [1]. The markers for this type are anti-liver kidney microsomal antibody type 1 (anti-LKM1) and anti-liver cytosol type 1 (anti-LC1). The prognosis for type 2 is poor compared to type 1. Young females are the primary target of this condition with a female-to-male ratio of about 4:1 [2]. The United States has a marginally higher prevalence of about 31.2 per 100,000 people compared to Europe’s 11-25 per 100,000 per year [2,3].

Tetracyclines are a major class of antibiotics used for a variety of clinical conditions such as rosacea, infections, and many other conditions. However, since their initial description in 1951, the hepatitis cases in patients taking this medication have been on the rise [4]. Standing out from the rest, minocycline is the most common cause of AIH in this class. The duration of usage is a key factor in further understanding the link between AIH and minocycline. As it pertains to our case, we will focus on its use in acne vulgaris. The question, "For how long are most patients on minocycline before AIH develops?", is where the gray area starts in our knowledge of this link. Most cases have been reported in the first two years of taking minocycline [5]. However, it is also possible for AIH to develop after just a few months of minocycline therapy. In our case, for example, AIH transpired in a matter of three months. Thus, it is safe to say that the duration of minocycline therapy is only one of the many variables that predisposes one to AIH.

Furthermore, although we know tetracyclines can cause AIH, it is important to understand the other etiologies as well to better understand why duration varies from case to case. Genetic conditions certainly can play a role. Autoimmune polyendocrinopathy-candidiasis-ectodermal dystrophy syndrome (APCEDS) is an inheritable condition involving AIH [1]. In addition to APCEDS, many patients with AIH present with co-existing autoimmune conditions such as diabetes mellitus type 1, Graves disease, and celiac disease, possibly due to the similar autoimmune dysfunction mechanism involved in both cases. Moreover, there are other genetic factors such as human leukocyte antigen (HLA) subtypes that lead to a greater predisposition in developing AIH. Although HLA DRB1*03 and HLA DRB1*04 can lead to AIH type 1, the former leads to a worse prognosis [1]. Such variables can certainly have an impact on when AIH will occur during minocycline therapy.

## Case presentation

A 16-year-old male presented to the emergency department with flu-like symptoms, one week of progressively severe abdominal pain and darker than usual urine. The pain was described as constant and deep. The patient reported taking tylenol for the pain and it helped reduce the pain from 9/10 to 2/10 on the pain scale. He denied any pain changes with positional movement. The pain radiated slightly to the flank region. The patient denied similar instances of this pain prior to this event. He has no significant past medical history aside from acne vulgaris. His family history was remarkable for diabetes mellitus type 2 and high blood pressure. His only medication included minocycline 50 mg, once a day, for his acne that he had been taking for about three months. He had no prior hospitalizations. A review of systems revealed headache, fatigue, and abdominal pain.

His vitals were normal except for a fever of 100.2 °F. A physical exam revealed a jaundiced patient in discomfort at bedside. Bowel sounds were normal and there was no rebound tenderness nor rigidity. The exam revealed a soft abdomen with tenderness in the right upper quadrant on deep palpation. Comprehensive blood work was remarkable for aspartate aminotransferase (AST) and alanine aminotransferase (ALT) elevations of 321 and 289 U/L, respectively. The total bilirubin measured about 5 mg/dL with a conjugated level of 2.8 mg/dL. The immunoglobulin levels were elevated with an immunoglobulin G (IgG) level of 35 g/L. ANA, anti-LKM1, anti-LC1 and anti-soluble liver antigen (anti-SLA) levels were all in range. The ASMA level was elevated at 1:110 titers. Acetaminophen levels were normal. A hepatitis panel was ordered that ruled out common viral etiologies. An ultrasound of the abdomen showed heterogeneous features on the liver with some nodularity throughout. A high-resolution CT scan revealed diffuse heterogeneous material on the liver. Magnetic resonance cholangiopancreatography (MRCP) revealed clear irregular-nodular patterns over the entire liver surface. Consequently, a biopsy was performed that demonstrated interface hepatitis along with bridging necrosis between portal tracts. The diagnosis of autoimmune hepatitis was made.

The rest of the hospital visit was uncomplicated. The patient was placed on prednisone therapy. His bilirubin was eventually normalized as well. He was later tested for HLA DRB1*03 and HLA DRB1*04 variants, but was found to be negative. He was educated on his condition and advised to stay away from minocycline. He was then weaned off of prednisone after the induction and maintenance phase, which was collectively 24 months. A repeat liver biopsy showed no hint of pathology reoccurrence. The patient’s liver function tests were monitored up to 10 years after the incident with no abnormal findings after treatment and instructions to steer clear of minocycline.

## Discussion

The risks for AIH can be separated into the following categories: age, gender, certain medications, and genetic factors. The highest incidence seems to occur in individuals who are 10-30 years of age and it occurs mostly in the female population. Although there are a number of common medications that could be the culprit, the most common are nitrofurantoin, halothane, and minocycline [1]. In our patient, a very odd observation can be made: he was on a low dose of minocycline and developed AIH in only three months. The time to develop AIH on minocycline therapy was significantly much less than the average of two years for most minocycline-induced AIH cases reported [5]. Aside from labs proving there was acute hepatitis, our leads to suspect AIH in this patient were the minocycline trial, ASMA as well as IgG elevation, and biopsy.

Generally, the presentation of AIH involves flu-like symptoms, abdominal pain, and jaundice. Our patient clearly presented with these. Other symptoms may present themselves while common symptoms may not always be there. Blood should be drawn to test for specific markers of AIH liver inflammation while differentiating other causes. Labs to look out for are abnormal AST, ALT, international normalized ratio, and bilirubin levels. The first two represent an obvious inflammatory process in the works, while the other two represent a declining functional issue of the liver. A viral hepatitis panel and the acetaminophen level should also be ordered to exclude other causes such as infectious or medication toxicity. As was done for our patient, antibody tests should also be ordered, such as ANA, ASMA, anti-LKM1, anti-LC1, anti-SLA, and total IgG. Among these, anti-SLA is a highly specific but poor sensitive AIH marker that is often used when the others are not positive [6]. Apart from labs and a physical exam, the gold standard for AIH diagnosis is a liver biopsy. In acute or late stages of AIH, most often the histopathology resembles interface hepatitis with or without bridging necrosis [7]. After all is obtained, the Simplified Autoimmune Hepatitis Score should be calculated for the patient. This score ranges from 0 to 8 and renders the diagnosis of AIH likely at a 6+ level [8]. Our patient had a score of 6, hence, prompting the diagnosis of AIH.

Deciding when to treat is crucial as better prognosis is often correlated with treatment being started at the right stage. Immunosuppressive therapy is the choice of treatment for AIH. However, this mode of treatment carries a vast array of side effects, including but not limited to the risk of opportunistic infections. Hence, treatment is advised when there are signs of disease progression to a serious stage by at least one of these characteristics: AST/ALT levels exceed 10 times the normal limit, AST/ALT level exceeds five times the normal limit coupled with an IgG level twice the normal limit, or a liver biopsy representing bridging or multiacinar necrosis on histology [1]. By virtue of the last characteristic, our patient qualified for immediate therapy. Prednisone, budesonide, and azathioprine are common medications used to treat AIH secondary to medications. Treatment should be individualized on a case-per-case basis and should last for a total of 24 months, including both induction and maintenance. A repeat biopsy of the liver should be taken that shows remission to begin weaning the patient off of steroid therapy.

One last point to be made about this case, in light of his negative HLA tests, is that it is unlikely that the rapid onset of AIH was brought about by genetic predisposition. The fact that he denied a family history of autoimmune diseases led us to one other likely predisposition category: environmental factors. These range from diet to chemical exposures. In the absence of common etiologies in this patient, it is logical to postulate that environmental factors likely played a role in the relative quick AIH development secondary to minocycline therapy.

## Conclusions

AIH is a complex condition with many environmental triggers as well as genetic predispositions. Our minocycline-induced AIH case was fortunate enough to respond to therapy because he was treated at the right stage when bridging necrosis had just started. Repeat liver biopsy after maintenance prednisone therapy showed total remission. A later follow-up after a decade showed no recurrence of AIH nor any residual liver dysfunction after the triggering agent was removed from his diet. Contrary to the two-year average that majority of cases present with, this article serves to shed light on the possibility of AIH occurring fairly quick in some patients taking minocycline. Although a rare complication, minocycline-induced AIH is still a potentially fatal complication that necessitates more frequent monitoring of liver functions of patients taking this medication.

## References

[1] Fialho Fialho, A. A., Fialho Fialho, A. A., & Carey, W. D. (n.d (2022). Autoimmune hepatitis. https://my.clevelandclinic.org/departments/digestive/medical-professionals/hepatology/autoimmune-hepatitis#treatment-tab .

[2] Linzay CD, Sharma B, Pandit S (2021). Autoimmune hepatitis. StatPearls [Internet].

[3] Tunio NA, Mansoor E, Sheriff MZ, Cooper GS, Sclair SN, Cohen SM (2021). Epidemiology of autoimmune hepatitis (AIH) in the United States between 2014 and 2019: a population-based national study. J Clin Gastroenterol.

[4] Glenn C, Feldman SR (2011). Tetracycline-induced hepatotoxicity. Dermatol Online J.

[5] Harmon EG, McConnie R, Kesavan A (2018). Minocycline-induced autoimmune hepatitis: a rare but important cause of drug-induced autoimmune hepatitis. Pediatr Gastroenterol Hepatol Nutr.

[6] Efe C, Ozaslan E, Wahlin S (2013). Antibodies to soluble liver antigen in patients with various liver diseases: a multicentre study. Liver Int.

[7] Geller SA (2014). Autoimmune hepatitis: histopathology. Clin Liver Dis (Hoboken).

[8] Hennes EM, Zeniya M, Czaja AJ (2008). Simplified criteria for the diagnosis of autoimmune hepatitis. Hepatology.

